# Changes in Health and Physical Fitness Parameters After Six Months of Group Exercise Training in Firefighters

**DOI:** 10.3390/sports8110143

**Published:** 2020-10-28

**Authors:** Matthew L. Sokoloski, Brandon R. Rigby, C. Ryan Bachik, Ryan A. Gordon, Isaac F. Rowland, Emily L. Zumbro, Anthony A. Duplanty

**Affiliations:** 1 School of Health Promotion & Kinesiology, Texas Woman’s University, Denton, TX 76204, USA; msokoloski@twu.edu (M.L.S.); rgordon4@twu.edu (R.A.G.); ezumbro@twu.edu (E.L.Z.); aduplanty@twu.edu (A.A.D.); 2 REACT Neuro-Rehab, Addison, TX 75001, USA; rbachik@neuroreaction.org; 3 Department of Health and Human Development, Montana State University, Bozeman, MT 59717, USA; ifrowland60@gmail.com

**Keywords:** group exercise, circuit training, flexibility, endurance, first responder, tactical

## Abstract

Proper training methods may be used as an effective preventative measure for many of the musculoskeletal injuries sustained as a first responder that are inherent to the profession. The traditionally low fitness levels and poor exercise habits of city firefighters may predispose this population to an increased risk of chronic conditions, such as cardiovascular and metabolic disease. The purpose of this study was to analyze changes in the health and fitness parameters of professional firefighters across North Texas during a six-month training program. Twenty-two professional firefighters completed six months of group training, consisting of two training sessions per week. These individuals underwent a pre- and post-fitness testing protocol that consisted of body composition, range of motion, anaerobic power, muscular endurance, and cardiorespiratory fitness. Improvements (*p* < 0.05) in flexibility, anaerobic performance, fatigue index, muscular endurance, and aerobic fitness were found following the six-month training program. No differences in body composition or peak power were observed (*p* > 0.05). Six months of group exercise improves aerobic and anaerobic fitness, exercise tolerance, muscular endurance, and flexibility in firefighters.

## 1. Introduction

Every year, cities budget hundreds of thousands of dollars for rehabilitation services that occur due to work-related injuries affecting first responders [1]. While many of the injuries sustained as a first responder are unavoidable due to the inherent risk of the profession, proper physical fitness and training methods may be an effective method in reducing injury risk. In the United States, the highest incidence rates for emergency responders’ injuries are soft tissue sprains and strains in the lower trunk, ankles, and knees [2]. Additionally, due to the unique physical demands of this profession, high levels of neck, back, and shoulder pain are also reported with this population [3].

The physical demands of firefighting require the men and women employed in this profession to be, at minimum, in good physical condition. Low fitness levels and poor exercise habits in firefighters predispose them to an increased risk of cardiovascular disease and obesity-related diseases [4]. Proper physical fitness may mitigate the risk of musculoskeletal injuries during job-related tasks in these individuals [5].

The need for improvements in the fitness levels and body composition of firefighters has recently been proposed by others [6]. Indeed, most departments throughout the United States do not mandate their first responders to exercise during their careers [6], and thus the health and fitness levels of firefighters can be overlooked in the absence of a yearly physical exam. Professional firefighters who possess high levels of aerobic fitness, anaerobic capacity, and muscular strength and endurance have increased mobility, energy, and endurance [7]. However, health-related measures of physical fitness, including aerobic capacity and maximum oxygen consumption (VO_2max_) [8,9], upper-body strength, and lower-body power [10], may decline with age in this population. Proper levels of physical fitness among firefighters may allow job duties to be performed more efficiently and safely, with those who are fitter less likely to jeopardize the safety of their fellow firefighters or the public they serve [7]. Despite this, mandating participation in a training program is not required for employment.

There is little published evidence regarding the benefits of long-term exercise training with tactical populations, more particularly with firefighters. This is likely due, in part, to the varying lifestyles of these professionals, aspects of which are difficult to control. Fitness standards among firefighters throughout the United States are mostly inconsistent, and research-based training programs are needed across departments. If exercise prescriptions are mandated between fire stations, improvements in physical fitness may accompany this programming, which may result in improvements with symptoms associated with acute and chronic conditions. If firefighters are more physically prepared to perform common tasks of their occupation, those tasks may be completed more safely and effectively. The purpose of this study was to analyze changes in the health and fitness parameters of professional firefighters across North Texas before and after a six-month group training program.

## 2. Materials and Methods

### 2.1. Participants

Thirty-four professional city firefighters (32 male, 2 female), aged 26 to 58 years, from the North Texas area were recruited. All individuals completed the physical activity readiness questionnaire (PAR-Q + 2019) and a medical history questionnaire prior to any data collection to confirm that no preexisting condition that would preclude them from exercise was present. The exercising habits of the participants at baseline widely varied between participants. Some of the firefighters could be considered recreationally fit, while others either did not adhere consistently to a structured exercise program or did not exercise at all. Written consent was provided prior to participation and all procedures were approved by the university’s institutional review board and participants were informed of the benefits and risks of the investigation prior to signing the informed consent.

### 2.2. Preliminary Testing Procedures

For each participant, all preliminary testing was conducted on one day and within 24 h after the participant’s most recent work shift. All testing procedures were conducted and supervised by trained research personnel who were CPR-/AED-certified. Upon arrival to the laboratory, participants were instructed to wear light exercise clothing (e.g., t-shirt, shorts, tennis shoes), eat breakfast at least 2 h prior to arrival, and refrain from consuming alcohol and caffeine over the previous 24 h. To begin, height was measured using a stadiometer (Perspective Enterprises, Kalamazoo, MI, USA), and weight was measured using a digital scale (Tanita, Arlington Heights, IL, USA). Body mass index (BMI) was calculated from these measures using the following equation:

(1) $$\mathrm{BMI}=\;\frac{\mathrm{mass}\;\left(\mathrm{kg}\right)}{{\mathrm{height}}^{2}\;\left(m^{2}\right)}$$

Body composition was then analyzed via dual energy X-ray absorptiometry (DXA; General Electric Lunar DXA-Prodigy, Madison, WI, USA). The DXA was calibrated at the start of the day’s procedures per the manufacturers’ recommendations using a QA (quality assurance) block phantom. Participants were instructed to lie motionless in a supine position, with their shoes and any wearable metals removed, for 6 min. The participants’ feet were strapped together. Next, peak power, mean power, and fatigue index were assessed with a 30-s Wingate protocol (Velotron, Quarq Technology, Spearfish, SD, USA), where participants pedaled at a maximal effort against 7.5% of their body weight (kg). Then, trunk and hamstring flexibility was measured using a Sit-and-Reach Test (Figure Finder Flex-Tester, Novel Products, Inc., Rockton, IL, USA). For this test, participants removed their shoes and sat with their feet flat against the box. Keeping their legs extended and their hands overlapped, the participants reached forward slowly and pushed the pin on the box as far forward as possible. The terminal position was held for 2 s and knees were extended throughout. Next, muscular endurance of the upper body and abdominals was assessed using the American College of Sports Medicine’s (ACSM) push-up and curl-up test, respectively. The push-up test was performed until the participant was no longer able to complete a full repetition. The one-minute curl-up test was performed with an upper limit of 70 repetitions using a metronome at 40 bpm. Finally, a graded exercise test (GXT) was conducted to measure cardiorespiratory fitness. This was performed using a Bruce Protocol on a motorized treadmill (Quinton Q Stress, Ventura, CA, USA). Respiratory gasses were continuously collected using an integrated metabolic system (ParvoMedics, Sandy, UT, USA) to measure VO_2max_. The metabolic system was calibrated at the start of the days’ procedures per the manufacturers’ recommendations, using both flow meter calibration and gas analyzer calibration. Participants were fitted with a mask and noseclip and were instructed to sit quietly for 5 min before the GXT began. At the start of the test, participants straddled the treadmill and then stepped on to the treadmill when ready. Maximum oxygen consumption was recorded if two of the following criteria were met: (1) an observable plateau in VO_2max_ was achieved; (2) a respiratory exchange ratio (RER) > 1.1 at volitional fatigue, or; (3) a heart rate at volitional fatigue within 10 beats of age-predicted maximum heart rate (calculated as 220-age) [11]. Maximal aerobic fitness was determined to as the highest VO2 value recorded during 30 s intervals.

### 2.3. Group Training Intervention

All training sessions were performed at REACT Neuro-Rehab (Addison, TX, USA). Each firefighter trained twice per week, and could attend any two sessions over a six-day period each week. However, all sessions typically started at 7:30 A.M. and lasted approximately 1 h. Due to the freedom in scheduling, a typical training session on any given day included six to eight participants. Sessions remained consistent in formatting, beginning with a dynamic warm-up, aerobic and resistance exercises, and a cool-down to conclude each session. A conjugate style periodization was chosen for this six-month plan. During the first four weeks of training, workouts were introduced, and all movements were taught, demonstrated, and practiced, ensuring the safety for all participants. All intervention protocols were conducted and supervised by two certified strength and conditioning coaches (CSCS) and tactical strength and conditioning facilitators (TSAC-F) through the National Strength and Conditioning Association (NSCA).

The group exercise included a combination of both aerobic and resistance exercises that were typically performed in a circuit style. During these workouts, participants typically completed as many repetitions in the allotted time, or attempted to complete the selected repetitions as fast as possible before moving to the next exercise. The strength coaches helped determine proper loads for each participant during each training session, were responsible for program design and auto-regulating training sessions based on the timing of the previous work shift and quality of sleep, and were tasked to modify training to ensure that all firefighters were able to complete all workouts. For example, if a push-up could not be performed, participants were instructed to perform push-ups with their knees on the ground. While these modifications and the autoregulation of training programs could affect each participant’s training load and volume, it was allowed in order to maintain ecological validity. A complete list of the workouts performed during the intervention, organized by day and week, is found in Table A1 in Appendix A. The participants were asked to maintain their typical dietary, sleep, and physical activity habits throughout the intervention.

### 2.4. Post-Testing Procedures

Following the six-month intervention, post-testing procedures were completed in the same manner as the preliminary procedures. All post-testing was completed within 7 to 10 days following the end of the training intervention.

### 2.5. Statistical Analysis

Four repeated-measures multivariate analyses-of-variance (MANOVAs) were used to examine differences between pre- and post-testing measurements. The first MANOVA grouped body weight, BMI, body fat percentage, and lean body mass. The second repeated-measures MANOVA grouped peak power, mean power, and fatigue index. The third repeated-measures MANOVA grouped the results of the push-up test, curl-up test, and flexibility assessment. The fourth repeated-measures MANOVA grouped the results of relative VO_2max_ and time to exhaustion during the Bruce protocol. Sidak post-hoc analysis was also used to examine whether significant effects were observed in all MANOVA tests. The level of significance was set at 0.0125 to avoid violating a type 1 statistical error (0.05/4). Cohen’s d effect sizes were reported and calculated as: d = (pretest score − posttest score)/pooled standard deviation. All statistical analyses were performed using SPSS statistical software (IBM SPSS Statistics v.24, Armonk, NY, USA).

## 3. Results

### 3.1. Participant Characteristics and Intervention Adherence

Thirty-four professional firefighters completed pre-testing for this study. Twelve participants were dropped from data collection as a result of not adhering to program protocol (i.e., did not participate in at least 75% of all training sessions, did not attend post-testing). Twenty-two participants (20 males and 2 females) completed all required procedures and are included in the final data analyses. Characteristics of these participants can be found in Table 1. There were no significant differences for weight, BMI, body fat percent, and lean mass between pre- and post-intervention.

### 3.2. Effects on Physiological and Fitness Measures

Differences between the pre- and post-intervention for cardiorespiratory fitness, anaerobic power, muscular endurance, and flexibility can be found in Table 2, Table 3 and Table 4. All fitness measures, with the exception of peak power improved following the six-month intervention. More specifically, there were increases in VO_2max_ (*p* = 0.005), and time to exhaustion (*p* = 0.012) during the GXT. Additionally, improvements in mean power (*p* < 0.001) and fatigue index (*p* < 0.001) during the Wingate protocol were observed following the six-month intervention. Improvements were also observed with the push-up (*p* = 0.005), curl-up (*p* < 0.001), and sit-and-reach tests (*p* < 0.001).

## 4. Discussion

The purpose of this study was to analyze changes in the health and fitness parameters of professional firefighters across North Texas before and after a six-month group exercise training program. The major findings from this study include improvements in anaerobic power, muscular endurance, flexibility, and cardiorespiratory fitness in firefighters following six months of group exercise training. These results may provide training guidelines to firefighters who wish to improve measures of their physical fitness. However, with an observed attrition rate of 35% in this study, exercise programming considerations (including mode, intensity, frequency, and duration) and training guidelines should be approached with caution if a national mandate for regular exercise training is implemented among firefighters in the U.S. Nevertheless, improved fitness levels may allow firefighters to perform their work-related duties more effectively and safely.

In this study, a heterogeneous sample of participants was intentionally recruited, which may accurately depict the range of fitness levels found in a typical fire station, as VO_2max_ values can vary widely between firefighters [12]. This method of recruitment, as opposed to recruiting a more homogenous group of participants with regard to (lack of) fitness level, could lead to an observed decrease in the potential magnitude of change in the measured variables. One example of this outcome is the measurement of BMI. Indeed, 9 of the 22 participants (41%) had a BMI over 30 kg/m^2^ at baseline in this study. This is comparable to data reported by Wilkinson et al. [13], who found that 29% of male firefighters were classified as obese according to BMI from a sample of 924 participants. If all participants were obese according to BMI, the observed magnitude of change of this variable may have been greater than what was reported in this study.

Lean body mass, total body fat percentage, and BMI were quantified before and after the six-month intervention in this study. Firefighters who have high body fat percentages typically score lower on ability assessment tests that are used to predict job-related performance [12]. An increase in muscle mass by 3% and a decrease in body fat by 2.7% was observed in the present study. The insignificance of the body composition findings may be explained by the lack of an implemented dietary intervention. Also, a lower frequency of exercise sessions (twice per week), performed throughout the training period, could have influenced the results. Indeed, improvements in body composition are greatest when exercise is combined with a dietary intervention high in protein [14].

Cardiorespiratory fitness was also measured using VO_2max_. Following the six-month training, there was a statistically significant increase (7.6%) in VO_2max_. These findings differ from to those reported by Cosgrove et al., who only found increases in VO_2max_ of approximately 0.7% in female participants and 3% in male participants following a high-intensity functional training (HIFT) protocol of similar length [15]. A reason for these lower values in the study by Cosgrove et al. could be that the authors did not track actual attendance for the prescribed interventions that required participants to exercise at least two days per week [15]. Interestingly, when relative VO_2max_ values are converted to absolute VO_2max_ values at pre- and post-test in this study, there appears to be a minimal change (4.4%) in aerobic capacity (3.41 L/min vs. 3.56 L/min, respectively). However, relative VO_2max_ is a more fundamental measure of job performance in firefighters when compared to absolute VO_2max_ [16], particularly when handling of added equipment and other supplementary devices inherent to the profession (e.g., hoses, ladders) is considered [10]. Time to exhaustion was also assessed during the GXT. Total time spent exercising during this test significantly increased (10.6%) post-intervention. This positive change of approximately 1 min may provide evidence of improved exercise tolerance.

A 30-s Wingate protocol was performed to determine the power output changes of participants over the course of the training period in the current study. Measures of peak power, mean power, and fatigue index are all indicators of anaerobic power and performance, and therefore capacity [17]. During certain job tasks, firefighters are required to have high levels of anaerobic capacity [7]. The anaerobic power measures in this study are an indicator of the ability to utilize anaerobic energy sources. Indeed, anaerobic energy sources are needed for bouts of strenuous on-duty tasks in the profession of firefighting [7], and anaerobic power correlates highly with firefighter performance [18]. In this study, a 7.1% increase in mean power, an 11.2% decrease in fatigue index, and a 3.3% increase in peak power was found post-intervention. Herbert et al. [19] reported a 7% increase in peak power following six weeks of high-intensity interval training (HIIT) training in endurance athletes. Similarly, Naimo et al. [20] reported a 10.9% increase in peak power, an 8.1% increase in mean power, and a 4.2% decrease in fatigue index following four weeks of HIIT in male collegiate ice hockey players. The improvements in peak power in Naimo et al. could be explained through the direct translation between HIIT training and its similarities to the Wingate assessment. This training directly mimics a typical shift in duration and intensity in the sport of ice hockey. The training intervention presented by Naimo et al., which implemented a true HIIT training protocol using short maximal-effort sprints on a bike interspersed with rest, differs from the exercise intervention presented in the current study [20]. The differences between these findings and the results of the current study may also be attributed to population and training frequency.

Muscular endurance was assessed using push-up and curl-up tests recommended by the ACSM [21]. Upper-body muscular endurance is strongly correlated with performance scores on Firefighter Ability Tests [12]. In the current study, a 20.7% and 118.2% increase in the number of push-ups and curl-ups completed, respectively, was observed following six months of group exercise. Our results were similar to La Reau et al. [22], who reported an increase of 27.2% in the number of push-ups completed using a similar testing protocol after 16 weeks of training in professional firefighters. In addition, the duration of a maximal-effort plank (a measure of core muscle endurance) improved by 89% in this same study [22]. The large increases in core strength may be due to high levels of lower-back pain, which limited five firefighters from completing any repetitions in the curl-up assessment pre-intervention. Following the intervention, there was an anecdotal decrease in lower-back pain. This may be due to an increase in the flexibility of the lower back and hamstrings, along with increases in muscular endurance of the core.

Flexibility of the lower back and hamstrings was analyzed in the present study due to the reported prevalence lower-back pain in professional firefighters [3]. Following our intervention, firefighters improved sit-and-reach scores by an average of 25.8%. In previous reports, flexibility outcomes are conflicting. Sobrero et al. [23] reported no significant differences in sit-and-reach scores in recreationally active women following six weeks of high-intensity functional training (HIFT) when performed three days per week. Hilyer et al. [24] found a reduction of workers’ compensation claims associated with joint-related injuries in firefighters following a training intervention focused on flexibility.

Our study presented unique differences from previously published studies using similar participants. Hollerbach et al. trained firefighters for 10 weeks via an online-based training program that included aerobic, body-weight, and weight-lifting components. This online training program differed by including not only a training program, but also education on nutrition and mental health. This study also differed in testing procedures. This research team tested changes in a simulated fire-ground test prior to, and following, the 10-week intervention [25]. A similar training pattern presented in the current study was utilized by Pawlak et al. However, the authors in this study included exercises incorporating equipment typically found in fire stations (e.g., sledgehammers, ladders, hoses), rather than traditional gym equipment (e.g., barbells, dumbbells) [26]. Due to the high prevalence of lower-back pain in firefighters, Mayer et al. implemented a 24-week endurance training program that utilized exercises targeting musculature in the core and lower back [27]. Roberts et al. recruited firefighter recruits, and tracked changes in performance measures throughout a structured exercise training program that included separate aerobic and resistive components. [28]. In summary, exercises within the workout plans presented in the current study were carefully chosen with the specificity of firefighting taken into consideration. This created unique programming considerations when designing the intervention protocol, which have differed from those presented in previous reports.

The most significant limitation in this study was the small sample size. Although 34 participants were recruited, there was a 35% attrition rate in this study. The final sample size of 22 participants was small, and that limited statistical power. With this small sample size, results (e.g., means, effect sizes) may not be reflective of the actual population of first responders. Also, another limitation to this study was the lack of a control group. A control group was not included in this study because we sought to encourage exercise participation among the potential participants. Included in this issue of program design was that the participants self-selected the weight used during the workouts, which made it difficult to control for intensity. No maximum strength assessment was conducted, so the intensity was mostly perceptive rather than objective in this study. This study also lacked an implemented nutritional intervention. Some firefighters in the current study made positive nutritional changes throughout the six-month intervention, while others chose to continue normal eating habits. Lastly, there was a lack of control in sleep patterns, which is typical with firefighters.

## 5. Conclusions

In conclusion, group exercise training appears to be a beneficial method of improving select health-related physical fitness measures in professional firefighters. Despite the lack of recording dietary habits, the absence of a control group, and the small sample size, increases in cardiorespiratory fitness, muscular endurance, flexibility, mean power, and fatigue index were observed in this study. When performed twice per week, there were no improvements in peak power or anthropometric measurements such as BMI, lean body mass, and body fat percentage. If a firefighter’s goal is to improve these measures by any significant amount, an adjustment to diet and number of training sessions per week may be required. Indeed, the implementation of a health program, including physical activity and dietary behavior interventions, may allow for a decrease in firefighter worker compensation (WC) claims and medical costs [29]. Moreover, the maintenance of a healthy body weight may reduce injuries and WC claims in firefighters [30]. More exercise training interventions with this population are therefore needed, as the eventual impact on worker compensation ($63.3 billion covered by worker’s compensation in 2012 [31]), or time off due to injury (30.6% of reported injuries resulted in lost workdays in 2016 [31]), is evident.

## Figures and Tables

**Table 1:** Characteristics of the participants before and after six months of group exercise.

Variable	Pre-I	Post-I	p-Value	Δ	Effect Size d
Age (years)	37.6 ± 10.0	-	-	-	-
Body mass (kg)	96.0 ± 7.9	93.1 ± 8.9	0.116	−3.0%	0.34
Height (cm)	180.0 ± 6.9	-	-	-	-
Lean Body Mass (kg)	66.0 ± 12.0	66.4 ± 11.3	0.058	−0.6%	−0.03
Body Fat (%)	29.3 ± 9.3	28.5 ± 7.5	0.147	−2.7%	+0.09
BMI (kg/m^2^)	30.0 ± 4.2	29.8 ± 4.1	0.524	−0.7%	+0.05

Note. All intervention values are presented as means ± standard deviation; BMI = body mass index (kg)/height (m^2^); Pre-I = pre-intervention; Post-I = post intervention; Δ = ([posttest score − pretest score]/pretest score) × 100; d = (pretest score − posttest score)/pooled standard deviation.

**Table 2:** Cardiorespiratory measures before and after six months of group exercise.

Variable	Pre-I	Post-I	*p*-Value	Δ	Effect Size d
VO_2max_ (ml/kg/min)	35.5 ± 4.9	38.2 ± 5.8 *	0.005	+7.6%	−0.50
Time to Exhaustion (min)	9.4 ± 1.0	10.4 ± 1.5 *	0.012	+10.6%	−0.78

Note. All intervention values are presented as means ± standard deviation; VO_2max_ = maximum oxygen consumption; Pre-I = pre-intervention; Post-I = post intervention; Δ = ([posttest score − pretest score]/pretest score) × 100; d = (pretest score − posttest score)/pooled standard deviation; * = significantly greater than pretest score (*p* < 0.0125).

**Table 3:** Anaerobic power measures before and after six months of group exercise.

Variable	Pre-I	Post-I	*p*-Value	Δ	Effect Size d
Peak Power (W)	1097.7 ± 245.3	1133.5 ± 237.5	0.354	+3.3%	−0.15
Mean Power (W)	660.2 ± 125.5	706.9 ± 125.2 *	<0.001	+7.1%	−0.37
Fatigue Index	60.8 ± 7.4	54.0 ± 9.3 ^†^	<0.001	−11.2%	0.81

Note. All intervention values are presented as means ± standard deviation; Pre-I = pre-intervention; Post-I = post intervention; Δ = ([posttest score − pretest score]/pretest score) × 100; d = (pretest score − posttest score)/pooled standard deviation; * = significantly greater than pretest score (*p* < 0.001); ^†^ = significantly less than pretest score (*p* < 0.001).

**Table 4:** Muscular endurance and flexibility measures before and after six months of group exercise.

Variable	Pre-I	Post-I	*p*-Value	Δ	Effect Size d
Flexibility (cm)	57.0 ± 14.7	71.7 ± 16.7 *	<0.001	+25.8%	−0.93
Push-Ups	29 ± 15	35 ± 16 *	0.005	+20.7%	−0.39
Curl-Ups	22 ± 16	48 ± 26 *	<0.001	+118.2%	−1.20

Note. All intervention values are presented as means ± standard deviation; Pre-I = pre-intervention; Post-I = post intervention; Δ = ([posttest score − pretest score]/pretest score) × 100; d = (pretest score − posttest score)/pooled standard deviation; * = significantly greater than pretest score (*p* < 0.0125).

## Appendix A

**Table A1:** Complete list of workouts for six months of group exercise.

Week	Day 1	Day 2
1 – Introduction	Dynamic Warmup	Dynamic Warmup
2 – Introduction	Goblet Squat 3 × 5 Pushup 3 × 10 Band Pull Apart	KB Swing 3 × 5 Banded Row 3 × 10 Farmers Carry 3 × 20 yds
3 – Introduction	Landmine Deadlift 3 × 5 Military Press 3 × 8 Plank 3 × 30 s	Jacob’s Ladder 3 × 30 s Beep Test ×1
4 – Introduction	Box Jumps 2 × 5 Trap Bar Deadlift 3 × 5 Side Plank 3 × 30 s	DB Bench Press 3 × 5 DB Row 3 × 8 Good Mornings 2 × 10
5	10 rounds @ 15 s each: Banded KB Swings Banded Good Mornings Farmers Walks	10 rounds @ 15 s each: Pushups Med Ball Depth Drop Toss Maximal-Effort Plank
6	8 rounds @ 15 s each: Tire Flips Sledge Hammer Alternating Hits Farmers Walk	Circuit, 3 rounds @ 60 s each: KB Swing Reverse Lunge & Press Plank
7	Plyometric Push-Up 3 × 10 Trap Bar Deadlift 5 × 5 SA Farmers Walk 1 × 120 s	Box Jump 5 × 5 Military Press 5 × 5 DB Row 6 × 10
8	3 rounds @ 60 s each: Tire Flips SA Farmers Walk Sledge Hammer Alternating Hits Sled Pull	4 rounds @ 120 s each: Jacob’s Ladder Beep Test ×1
9	3 rounds @ 120 s each: Banded KB Swing Jacob’s Ladder	Military Press 5 × 8 Prone Row 4 × 12 Beep Test ×1
10	3 rounds @ 120 s each: DB Step-Up Side Plank	6 rounds @ 30 s each: Banded Row Push-Ups
11	DB Incline Press 5 × 5 AMRAP (10 min limit) BB Deadlift ×5 Inverted Row ×5 Push-Ups ×5	Trap Bar Deadlift 5 × 5 AMRAP (10 min limit) BO DB Row ×5 DB Military Press ×5 Med Ball Slam ×5
12	Landmine Deadlift 5 × 12 Good Mornings 4 × 8 Beep Test ×1	Landmine Press 5 × 12 DB Row 5 × 8 3 × 400 m run
13	6 rounds @ 30 s each: Tire Flips Sledge Hammer Alternating Hits Farmers Walk	Beep Test ×2
14	AMRAP ×2 (5 min limit each) DB Sumo Squat ×8 DB Bent Over Row ×8 Trap Bar Deadlift ×8 Farmers Walk (20 yds)	AMRAP × 2 (5 min limit each) Push-Ups ×8 Banded Row ×10 Band Pull Apart ×15 Med Ball Slam ×8
15	6 rounds @ 30 s each: DB Sumo Squat ×8 DB Bent Over Row ×8 Trap Bar Deadlift ×8 Farmers Walk (20 yds)	6 rounds @ 30 s each: Push-Ups Banded Row Band Pull Apart
16	6 rounds @ 30 s each: Landmine Deadlift Landmine Press Sled Drag DB Bench Press	6 rounds @ 30 s each: Goblet Squat DB Push Press Banded Row Battle Ropes
17	3 rounds @ 60 s each: Tire Flips SA Farmers Walk Sledge Hammer Alternating Hits Sled Pull	4 rounds @ 120 s each: Jacob’s Ladder Beep Test ×1
18	10 rounds @ 15 s each: Banded KB Swing Banded Good Mornings Farmers Walks	10 rounds @ 15 s each: Push-Ups Med Ball Depth Drop Toss Maximal-Effort Plank
19	3 rounds @ 120 s each: Banded KB Swing Jacob’s Ladder	Military Press 5 × 8 Prone Row 4 × 12 Beep Test ×1
20	3 rounds @ 120 s each: DB Step-Up Side Plank	6 rounds @ 30 s each: Banded Row Push-Ups
21	DB Incline Press 5 × 5 AMRAP (10 min limit) BB Deadlift ×5 Inverted Row ×5 Push-Ups ×5	Trap Bar Deadlift 5 × 5 AMRAP (10 min limit) BO DB Row ×5 DB Military Press ×5 Med Ball Slam ×5
22	Landmine Deadlift 5 × 12 Good Mornings 4 × 8 Beep Test ×1	Landmine Press 5 × 12 DB Row 5 × 8 3 × 400 m run
23	6 rounds @ 30 s each: Tire Flips Sledge Hammer Alternating Hits Famers Walk	Beep Test ×2
24	6 rounds @ 30 s each: Tire Flip Sled Pull Alternating Sledge Hammer Hits	6 rounds @ 30 s each: Push-Ups Banded Row Band Pull Apart
25	3 rounds @ 20 s each: Push-Ups Banded Row Goblet Squat Med Ball Slams	Beep Test ×1

Note. AMRAP = As Many Reps As Possible; BB = Barbell; BT = Beep Test; DB = Dumbbell; BO = Bent Over; KB = Kettlebell; SA = Single Arm.
